# Supplementary material for: Pilot study on the value of Raman spectroscopy in the entity assignment of salivary gland tumors
Source: PLoS One. 2021 Sep 16;16(9):e0257470. doi: 10.1371/journal.pone.0257470 (PMC8445432; doi:10.1371/journal.pone.0257470)
Supplement: S1 Fig — (DOCX) [file pone.0257470.s001.docx]

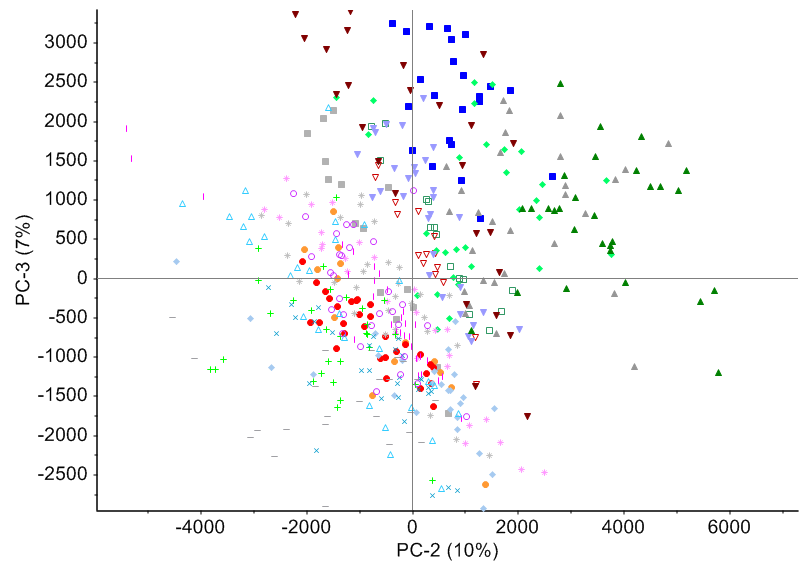


**S 1 Scores clustering dependent on the individual tumor samples:** Showing the scores of PC-2 and PC-3, there is a clustering in dependence of the different tumor samples visible.
