# Supplementary material for: Pilot study on the value of Raman spectroscopy in the entity assignment of salivary gland tumors
Source: PLoS One. 2021 Sep 16;16(9):e0257470. doi: 10.1371/journal.pone.0257470 (PMC8445432; doi:10.1371/journal.pone.0257470)
Supplement: S2 Fig — A good separation in dependence of the tumor entity is possible. (DOCX) [file pone.0257470.s002.docx]

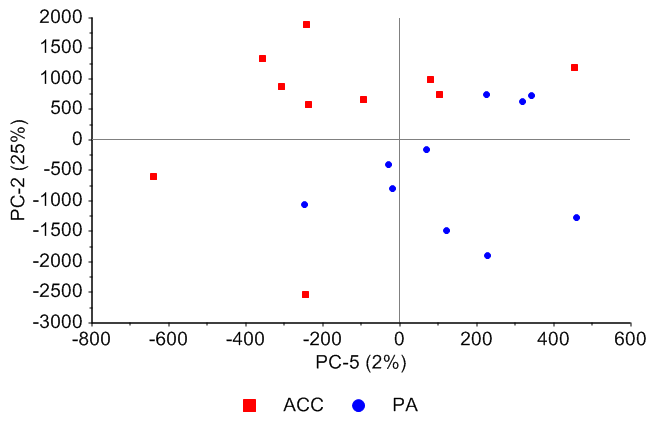


**S 2: PCA-recalculation with the PC-2 and PC-6 high and low peaks:** Scores plot after recalculation with the loading peaks bigger than 0.1 and smaller than -0.1 of the PC-2 and PC-6. A good separation in dependence of the tumor entity is possible.
