# Supplementary material for: Pilot study on the value of Raman spectroscopy in the entity assignment of salivary gland tumors
Source: PLoS One. 2021 Sep 16;16(9):e0257470. doi: 10.1371/journal.pone.0257470 (PMC8445432; doi:10.1371/journal.pone.0257470)
Supplement: Attachment — Submitted filename: Response-to-Reviewer.docx [file pone.0257470.s004.docx]

Yihong Wang

Academic Editor

PLOS ONE

|  | Dr. med. Till Meyer |
| --- | --- |
|  |  |
|  |  |
| Telefon | 0049 (0)931 201-21323 - Sekretariat |
| Telefax | 0049 (0)931 201-21321 |
| E-mail | meyer_t2@ukw.de |
|  |  |
|  |  |
| Würzburg, | 18.06.2021 |

**Reviewer #1:** The aim of this study is to evaluate the suitability of Raman spectroscopy for entity assignment in salivary gland tumors. In a principal component based linear discriminant analysis, majority of tumor samples are classified correctly. The topic of this paper is meaningful and interesting. However, there are still some concerns need to be addressed.

1. The readability of the article needs to be improved. The proportion of text narratives seems to be somewhat large. It is recommended that the authors simplify the text and add some charts to vividly describe the phenomenon revealed by the data.
*Thank you for catching this point. We revised the text passages and added some.*

2. As mentioned in the previous comment, it is recommended that authors supplement a flowchart and a mind map to better present their paper.
*Thank you for this further idea. We added a flow chart, that presents the tissue preparation steps.*

3. The resolutions of the figures in this paper need to be improved.
*We have enlarged the resolution of all figures.*

4. At the bottom of the Introduction, the authors need to summarize the remainder of the paper.
*We would like to address this concern. However to our best knowledge the submission guidelines of PLOS ONE does not contain a “remainder”.*

5. In the Materials and Methods section, the authors could add a pipeline diagram for tissue sampling and data processing to facilitate readers to better understand the paper.
*We added a diagram for the description of the tissue sampling to the manuscript (figure 1).*

6. In Raman spectroscopy section, a figure illustrating the tumor-specific areas may be helpful and a comparison of a healthy area also needs to be provided.
*This information is included in the new figure 1.*

7. The authors need to add a separate section to briefly introduce the principle of Raman measurement and the principle of measuring instrument. At this time, it would be better to provide some schematic diagrams.
*Thank you for catching this point. We added a section to the introduction and the new figure 1.*

8. More details of the seven principal components should be given.
*We added more information about all the different principal components in the new figure 4.*

9. The experimental results and discussions need to be enriched.
*According to point 8 we added additional results and also added more details to the discussion. Furthermore the recalculation data with the high peaks of PC-2 and PC-6 and a PCA with all single spectra were added for enrichment of the results.*

**Reviewer #2:** The authors applied Raman spectroscopy to discriminate pleomorphic adenomas (PA) and adenoid cystic carcinomas (ACC) for assessment of salivary gland tumors. Although Raman spectra of rare samples are very valuable, this reviewer thinks that the authors need to explain and evaluate properly their methods and conditions of the analysis.
*We addressed this concern by adding new figures, which show in detail, who the tissue processing and Raman measurement procedure.*

The authors seem to estimate the accuracy of their discrimination model according to the results of test datasets that are used to build the discrimination model itself. The reliability of the analytical model must be evaluated with the totally independent datasets. The number of spectra may not be enough to keep the test and validation datasets independently, because the sample is too rare. In such a case, leave-one-out cross validation method should be applied at least.
*For leave-one-out cross validation we calculated 20 different LDA-models with leaving every time one sample out. The left out sample we predicted with the remaining data set*. The overall accuracy of the model was 80%.

| Confusion matrix of LOO-PCA-LDA model with an overall accuracy of 80%. | | | |
| --- | --- | --- | --- |
|  | | True labels | |
|  |  | PA | ACC |
| Predicted labels | PA | 9 | 1 |
|  | ACC | 3 | 7 |

The authors must describe spectral treatment in detail. The spectra in Fig. 2 seems to be processed by baseline correction and/or background subtraction. The background spectra could give effect to the results of multivariate analysis.
*In deed baseline correction was performed by the use of the customized software of the Raman spectroscopy system. We added this information to the method section. Thank you for this valuable advice.*

This reviewer does not understand how the authors avoid the noise arising from paraffin. The authors describe “We addressed this limitation by using as unified deparaffinizing conditions as possible.” Commercial paraffine products are usually consist of multiple paraffine species. They have different characters in adsorption to the materials in tissue, which often give problems in erasing paraffine noise in the Raman analysis of paraffine fixed tissues. The authors must explain in detail.
*We addressed this by the use of a deparaffination protocol. However there will be still a rest of paraffin and furthermore especially fat fraction get washed away. But all samples were treated with the same fixation and deparaffination protocol, so that the conditions in term of paraffin-contamination should be comparable between the different samples. To our point of view the question in which way the biological Raman spectral information of the tissue is influenced by paraffination and deparaffination is important for development of RS applications in the future. Therefore we will address this question in detail in a upcoming project.*

The PCA seems to be applied to the averaged spectra consisting of 30 spectra each. However, the spectra of tumor-free areas, nerves, muscles, and blood vessels are excluded. The authors described that one of the major issues is the pre- or intraoperative determination of the tumor entity. Is it feasible to avoid those tissues visually without HE staining? How large it the focus volume of the Raman microscope? Do authors think that use of Raman probe which usually large focus volume is not suitable?
*To our point of view the tumor identification in HE-sections is definitely important. Therefore the co-registration of histology and spectroscopic evaluated slides is absolutely necessary and was performed in our study. HE-staining is also for frozen sections possible. Therefore we evaluate in a current study the use of consecutive frozen sections, native for spectroscopic measurements and HE stained for tumor identification. Only by histological tumor identification is a reliable tumor identification possible. There are research groups, that publish the use of Raman spectroscopy with a large focus volume, which was directly applicated to the macroscopic identified tumor surface. However to the authors opinion this approach is interesting, but the missing micromorphological control makes the accidental measurement of non-tumor structures more likely. This will enlarge the variation and therefore is the further development to a clinical translation more unlikely. To the authors view a translation to clinic of spectroscopic approaches is only realistic by the use in addition to the well-established classical histopathological analysis.*

The spectra should include a band near 1740 cm-1 due to the C=O stretching mode to distinguish fat and lipid.
*Indeed there is a discussion about the size of the biological relevant wavenumber range. It is common to include the wavenumber range from 1800 cm-1. We recalculated with the wavenumber range from 1800-600 cm-1. Including the wavenumber range 1800-1700 cm-1 had no impact on the model accuracy.*

**Reviewer #3:** In this contribution, the authors present a pilot study to use Raman spectroscopy for the entity determination in salivary gland tumors. Raman spectroscopy from de-paraffinized sections of pleomorphic adenomas (PA) and adenoid cystic carcinomas (ACC) samples (10 each) were measured and analyzed using principle component analysis (PCA). Classification accuracy of about 90% was achieved separating the spectrum from the two groups. While interesting, the current study has several major limitations, which needs to be properly addressed.

1. The clinical significance of the proposed Raman spectroscopy measurements is not clear or convincing. The authors tried to make a case for speed and use Raman “for quick intraoperative diagnosis or to improve the FNAC accuracy.” However, the argument is not convincing, as the samples need to be sectioned and Raman measurements are not fast. The experiments conducted were using paraffin fixed samples and does not reflect the proposed clinical use scenarios. The authors are suggested to use fresh or frozen section samples to demonstrate the feasibility.
*In deed the chosen experimental design by using deparaffinized tissue does not reflect the pursued setting to 100 %. However for a first evaluation, if RS is principally suitable for salivary gland tumor entity determination, we consciously have chosen deparaffinized tissue samples. The main reason is, that malignant salivary gland tumors are too rare to collect enough tissue samples in an adequate time, also in a big tertiary universal hospital with a very high case load. Therefore there is to our point of view no other way, to address this important research question in rarer diseases like salivary gland cancer.*

2. The number of specimens used for each group is very limited (10 each). Also, there is no control groups from healthy salivary gland samples. The authors acknowledged these limitations, but did not include more samples to address these issues.
*The main reason for the limited number of specimens is listed above. Usually spectroscopy approaches in cancer science were used for cancer detection. But in salivary gland tumor treatment is opposed to the classical head and neck squamous cell carcinoma treatment, the main challenge the entity and dignity determination but not the cancer border detection. Therefore in this clinical context healthy salivary gland tissue is not the relevant control group.*

3. Tissue heterogeneity may also affect the measurements and classification accuracy. How to better control this in clinical settings? The authors mentioned to use H&E slide to select only the tumor regions for analysis. But what about “quick intraoperative diagnosis” if H&E still needs to be prepared?
*Thank you for catching this point. HE staining is also in frozen sections possible. In a future study we address this problem by using consecutive frozen sections, native for spectroscopic measurements and HE stained for tumor identification. To the authors point of view the use of RS for in-vivo measurement is unrealistic, because of so many uncontrollable variables. The chance of translation innovative methods like RS to the clinic is much more probably in supporting existing and standardized clinical procedures like intraoperative frozen sections.*

4. Can images be generated from the Raman measurements and co-registered with histology or immunohistology slides? They may be more informative.
*To our point of view the tumor identification in HE-sections is definitely important. Therefore the co-registration of histology and spectroscopic evaluated slides is absolutely necessary and was performed in our study.*

**Reviewer #4:** The article is interesting and generally well written. The authors identify their limitation of not having normal salivary glad tissue characteristics in the model which could diminish the importance of their research work. If the reason of not having a normal tissue group is due to lack of samples, it's better to mention that in the article, or else if you have acquired samples after the manuscript submission then you could include those in the revised manuscript. My major concern with the article is that the authors mention that they collected 30 Raman spectra per sample which is reasonable for a pilot study and they have 20 samples in the study which should add up to 300 spectra for each group in the Figure 3 PCA plot but I don't see that. Did you average the spectra into one spectrum before doing the statistical analysis? If so, that will mask the spread of the data, the heterogeneity of the tissue that would be important to readers to see how each spectrum behave in the PCA space and understand it. The sample size is limited if you average by the sample and then do the analysis, unless you add a lot more samples, the conclusions drawn from the study may not be meaningful.
*Thank you for catching these important points. In deed we used average spectra for the model calculation. The reason for that approach is, that by building the average spectra a kind of spectra normalization is possible. To address your concern we added the PCA with the single spectra (see S1). The labeling is dependent of the sample number. A clustering along the sample number is visible.
Due to the rarely incidence of malignant salivary gland tumors, an inclusion of many more samples is not possible, also in a big tertiary high volume universal hospital.*
