# Supplementary material for: Pilot study on the value of Raman spectroscopy in the entity assignment of salivary gland tumors
Source: PLoS One. 2021 Sep 16;16(9):e0257470. doi: 10.1371/journal.pone.0257470 (PMC8445432; doi:10.1371/journal.pone.0257470)
Supplement: Attachment — Submitted filename: Response_to_Reviewers.docx [file pone.0257470.s005.docx]

Yihong Wang

Academic Editor

PLOS ONE

|  | Dr. med. Till Meyer |
| --- | --- |
|  |  |
|  |  |
| Telefon | 0049 (0)931 201-21323 - Sekretariat |
| Telefax | 0049 (0)931 201-21321 |
| E-mail | meyer_t2@ukw.de |
|  |  |
|  |  |
| Würzburg, | 27.08.2021 |

Dear Mr. Wang,

thank you for the detailed review and the helpful comments of the reviewers. By addressing the reviewers´ concerns, the quality of the manuscript could be improved significantly. Attached you can find the answers to all reviewers comments.

On behalf of all authors

Till Meyer

***Reviewer #1:*** *Some of my concerns have been tackled, but I am still unsatisfied with the presentation and solidity of this manuscript. Here are some remaining questions in the last review and new concerns.
1. The presentation of this manuscript is improvable.
- The proportion of text narratives still seems to be large.*
Thank you for this advice. We adjusted the manuscript and reduced the narratives.
*- The resolutions of the figures can be improved further.*
We have further improved the figure resolution and take care for the journal specific adjustments.

*2. This paper is not solid in theory and the experimental results are failed to support their claim.
- The experimental results and discussions are not enough.*
The claim of the manuscript is to basically proof that there is an entity dependent difference in in RS-signature. To our best knowledge, there is no other published manuscript that focusses on the question, if entity assignment in salivary gland cancer is in general possible. Our results clearly show that there is an entity dependent RS signature, focusing on differences in the mean spectra and multivariate data analysis. To our point of view we define important steps, that are really necessary to evaluate the possibility of clinical translation. Our study implements the RS technology and reliable proof of concept data with a clear focus on future clinical application, which is often neglected on other studies.  *- As other reviewers said the clinical significance of the proposed Raman spectroscopy measurements is not convincing.*
We regret, that we can´t carve out the clinical significance of the proposed Raman spectroscopy measurements. We adjusted the introduction. The clinical significance of the problem with the entity assignment in salivary gland tumors is clearly obvious for all clinicians who are immediately involved in the surgery therapy of salivary gland cancer. However, the clinical significance should be very obvious also for non-clinicians after reading the manuscript. Therefore, we restructured the introduction.
*- The sample size is not enough to support the results.*
Focusing on salivary gland cancer the incidence is low. However, also in a big tertiary care hospital much bigger number of cases is unrealistic. But to our point of view there is a need also to focus on tumor entities with low incidence. Comparing to other studies dealing with salivary gland cancer, the sample size is similar (e.g. Brozek-Pulska et al.). Please take in mind, that every patient is for us only one sample, a big difference to other manuscripts. If we focussed on our main tumor entities like squamous cell carcinoma a sample size of hundred and more would be managable.

***Reviewer #2:*** *The authors have not improved their manuscript sufficiently. It includes lots of inaccurate results and discussions. Although their data is valuable, the manuscript must be revised well before publishing.

The authors must describe how they made the baseline correction in detail and validate correctness of the process. Especially in multivariate analysis of Raman spectra, one must pay a big attention to the selection of background spectra. If one used 2 background spectra for correction of datasets and the datasets were categorized into 2 groups, the result could merely reflect the patterns of the background spectra. The authors must carefully explain how they avoid possible mistakes.*
Thank you very much for addressing this point. Indeed, the baseline correction can have a big impact on the results. We have performed the baseline correction with the data-export for every single patient sample independently. Therefore, there should be no influence on later performed entity assignment within the multivariate data analysis. *The PCA seems to be applied to the averaged spectra consisting of 30 spectra each. Their discrimination model could be applied only to the averaged spectrum of 30 spectra and could not be applicable to analysis of each Raman spectrum. Consequently, the value of the accuracy of their model, 90% that the authors describe, does not correct for a single spectrum.*
We totally agree with your statement. But it was not the aim, to validate the model for single spectra. Focusing on the clinical point of view, we used average spectra to reduce the power of single measurements respectively single spectra. We added this important point to the discussion. *The accuracy of a discrimination model must be evaluated with an independent dataset. In case that the number of data is too few to keep some data out for validation, one may use leave-one-out cross-validation method. As the author describe in the rebuttal letter, the overall accuracy to the averaged 30 spectra is 80%. It is not 90%.*
Indeed, the highest level of validation is the use of an independent dataset. To our point of view, this should be the next step. We performed leave-one-out cross-validation. The overall accuracy is in this model 80% and therefore lower than 90% in the in the LDA model. However, the leave-one-out cross-validation confirmed, that in general an entity assignment based on Raman spectra data is possible. *The bands in the spectra must be correctly labelled. In Table 2, a band at 1003-989 cm-1 is assigned to phenylalanine. However, phenylalanine always shows 2 bands near 1003 and 1030 cm-1. In contrast, fat species often show a sharp single band near 990 cm-1 and the spectra in Fig. 3 looks similar to their spectral pattern. The authors must show the spectral area from 1800 cm-1 to reveal their assignment.*
Thank you for this advice. We changed figure 3, to show the spectral area from 1800 cm-1 and corrected the labeling.

**Reviewer #3: (No Response)**
